# Editorial: Exploring gut neuroimmunology: focus on the enteric nervous system in health and disease

**DOI:** 10.3389/fnins.2025.1726789

**Published:** 2025-11-17

**Authors:** Matilde Otero-Losada, Sukhada Bhave, David Dora

**Affiliations:** 1 Centro de Altos Estudios en Ciencias Humanas y de la Salud, Universidad Abierta Interamericana, Consejo Nacional de Investigaciones Científicas y Técnicas (CAECIHS.UAI-CONICET), Buenos Aires, Argentina; 2 Columbia Technology Ventures, New York, NY, United States; 3 Semmelweis University, Budapest, Hungary

**Keywords:** gut-brain axis, neuroimmunology, enteric nervous system (ENS), microbiota, health and disease

The gut-brain axis represents a complex communication network linking the gastrointestinal (GI) tract and the central nervous system (CNS). A core component of this system is the enteric nervous system (ENS), often referred to as the “second brain,” which autonomously governs gut functions and interacts with the gut microbiome, mucosal immunity, and the autonomic nervous system. Recent research highlights the significance of microbiome-host interactions, gut permeability, and the integrity of pivotal barriers, including the gut epithelial, blood-nerve, and blood-brain barriers in maintaining neurological health. Dysregulation in these systems has been implicated in various neurodegenerative diseases, including Parkinson's disease (PD). This warrants further research and systematic integration of knowledge concerning gut neuroimmunology and gut-brain communication. This field holds transformative potential for translational and clinical science, promising novel therapeutic approaches.

This Research Topic aims to clarify the potential mechanisms underlying the gut-brain axis, focusing particularly on the enteric nervous system contribution in health and disease. We seek to uncover how disruptions in gut permeability and gut-brain signaling contribute to various neurological disorders by addressing gaps in the knowledge of gut microbiome dynamics, mucosal immunity, and their interplay with the ENS in neurodegenerative diseases like PD in particular. Recent advances in gut neuroimmunology and microbiome research offer encouraging opportunities for exploring these interactions. Four studies were selected for this Research Topic as follows.

*Neurofunctional correlates of emotional dysregulation in adolescent Crohn's disease: a resting-state fMRI preliminary investigation* (Liu et al.). The authors studied the relationship between abnormal intrinsic brain function and emotional symptoms in adolescent Crohn's disease (CD) patients using resting-state functional magnetic resonance imaging (rs-fMRI). The Inflammatory Bowel Disease Questionnaire (IBDQ), Symptom Checklist-90 (SCL-90), Social Support Rating Scale (SSRS), and resting-state functional magnetic resonance imaging (rs-fMRI) scans were used to evaluate 93 participants (40 adolescent CD patients and 53 healthy controls (HCs). Neuroimaging findings, clinical indicators, and psychometric measures were analyzed. Functional connectivity analysis revealed distorted patterns in specific brain areas, suggesting disease-specific hyperconnectivity in sensory-cognitive networks. The outcome of this study offers new insights into the neurobiological basis of emotional symptoms in adolescent CD patients, highlighting altered activity in temporal, frontal, and cerebellar regions (Butwicka et al., 2019; Cooney et al., 2024).

*Gut microbiota of patients with post-stroke depression in Chinese population: a systematic review and meta-analysis* (Li et al.). Evidence of changes in the composition and function of the gut microbiota (GM) in post-stroke depression (PSD) patients is gradually accumulating. This study evaluated the relationship between PSD and GM based on the Preferred Reporting Items for Systematic Reviews and Meta-analyses (PRISMA) criteria. The authors searched in PubMed, Web of Science, Embase, Cochrane databases, Wangfang, VIP, CBM, and CNKI from the establishment of the database to April 17, 2024. Systematic review and meta-analysis were performed to investigate the differences of GM between patients with PSD spectrum and healthy controls (HC) or stroke spectrum. The outcome provides relevant information on the relative abundance of Bacteroidota, Fusobacteriota, Pseudomonadota, and Bacillota in PSD patients and in the HC group, even at the family and genus levels (Simpson et al., 2021; Socała et al., 2021).

*Plasma exosomal miRNA expression and gut microbiota dysbiosis are associated with cognitive impairment in Alzheimer's disease* (Lin et al.). Alzheimer's disease (AD) patients differ from normally cognitive subjects regarding gut microbiota composition and expression profiles of microRNAs (miRNAs) in brain tissue, cerebrospinal fluid, and blood. This study explored the relationship between plasma exosomal microRNAs, gut microbiota, and cognitive impairment to provide insights into AD pathogenesis and treatment. A small sample of AD patients and subjects with normal cognition entered the study. The Mini-Mental State Examination (MMSE) allowed to evaluate cognitive function. High-throughput sequencing was used to identify differentially expressed miRNAs in plasma exosomes. Metagenomic sequencing detected potential differences in gut microbiota abundance. Interesting associations and differences were found in MMSE scores, the abundance of potential probiotics (*Faecalibacterium prausnitzii, Roseburia intestinalis*, and *Roseburia inulinivorans*) and specific exosomal miRNAs (miR-3120-3p, miR-6529-5p; miR-3120-3p, miR-6529-5p, miR-124-3p, and miR-3120-3p) between the two groups (Sheng et al., 2021, 2022).

*Exploring the relationship between GBA1 host genotype and gut microbiome in the GBA1 L444P/WT mouse model: Implications for Parkinson disease pathogenesis* (Menozzi et al.). Heterozygous glucosylceramidase beta 1 (GBA1) variants are the most frequent genetic risk factor for Parkinson's disease (PD), but penetrance is incomplete. GBA1 dysfunction was associated with gastrointestinal disturbances and microbiome changes in preclinical models. Growing evidence suggests that the microbiota-gut-brain axis may be involved in PD pathogenesis. Whether the host GBA1 genetics in heterozygosis influences gut microbiome composition has not been explored. The authors evaluated whether heterozygosity for the GBA1 pathogenic L444P variant could cause perturbations in gut microbiome composition. Based on their findings, they suggest that studies investigating the effect of a second hit on gut physiology and microbiome composition could explain the partial penetrance of GBA1 variants in PD (dos Santos et al., 2024; Stoker et al., 2020).

Collectively, these contributions emphasize the critical role of gut neuroimmunology in modulating neurological and psychiatric health. By integrating advances in neuroimaging, microbiome science, and molecular profiling, this Research Topic underscores the ENS as a key mediator of gut–brain communication and highlights novel pathways for therapeutic exploration. The selected papers sketch an attractive systems map from the lumen to the ENS neural cells, implying that host genotype, exosomal signaling, and microbiome ecology in longitudinal designs will be pivotal for translation. In other words, the ENS may be the first interface and best model to study where neurology meets ecology.

## References

[B1] Butwicka A. Olén O. Larsson H. Halfvarson J. Almqvist C. Lichtenstein P. . (2019). Association of Childhood-Onset Inflammatory Bowel Disease with Risk of psychiatric disorders and suicide attempt. JAMA Pediatr. 173, 969–978. doi: 10.1001/jamapediatrics.2019.2662 31424531 PMC6704748

[B2] Cooney R. Tang D. Barrett K. Russell R. K. (2024). Children and young adults with inflammatory bowel disease have an increased incidence and risk of developing mental health conditions: a UK population-based cohort study. Inflamm. Bowel Dis. 30, 1264–1273. doi: 10.1093/ibd/izad169 37603846 PMC11291622

[B3] dos Santos J. C. C. Mano G. B. C. da Cunha Barreto-Vianna A. R. . (2024). The Molecular impact of glucosylceramidase beta 1 (Gba1) in Parkinson's disease: a new genetic state of the art. Mol. Neurobiol. 61, 6754–6770. doi: 10.1007/s12035-024-04008-8 38347286

[B4] Sheng C. Lin L. Lin H. Wang X. Han Y. Liu S.-L. (2021). Altered gut microbiota in adults with subjective cognitive decline: the SILCODE study. J. Alzheimers. Dis. 82, 513–526. doi: 10.3233/JAD-210259 34024839

[B5] Sheng C. Yang K. He B. Du W. Cai Y. Han Y. (2022). Combination of gut microbiota and plasma amyloid-β as a potential index for identifying preclinical Alzheimer's disease: a cross-sectional analysis from the SILCODE study. Alzheimers. Res. Ther. 14:35. doi: 10.1186/s13195-022-00977-x 35164860 PMC8843023

[B6] Simpson C. A. Diaz-Arteche C. Eliby D. Schwartz O. S. Simmons J. G. Cowan C. S. M. (2021). The gut microbiota in anxiety and depression - a systematic review. Clin. Psychol. Rev. 83:101943. doi: 10.1016/j.cpr.2020.101943 33271426

[B7] Socała K. Doboszewska U. Szopa A. Serefko A. Włodarczyk M. Zielińska A. . (2021). The role of microbiota-gut-brain axis in neuropsychiatric and neurological disorders. Pharmacol. Res. 172:105840. doi: 10.1016/j.phrs.2021.105840 34450312

[B8] Stoker T. B. Camacho M. Winder-Rhodes S. Liu G. Scherzer C. R. Foltynie T. . (2020). Impact of GBA1 variants on long-term clinical progression and mortality in incident PD. J. Neurol. Neurosurg. Psychiatry 91, 695–702. doi: 10.1136/jnnp-2020-322857 32303560 PMC7361014

